# Halotolerant plant growth-promoting bacteria isolated from *Opuntia dillenii* (Ker Gawl) Haw (Cactaceae) may enhance tomato growth under salinity stress in greenhouse conditions

**DOI:** 10.1007/s00203-026-05229-2

**Published:** 2026-09-26

**Authors:** Yves Kévin Brun, Agossou Damien Pacôme Noumavo, Julien Colombet, Sibel Turali, Lamine Baba-Moussa, François Lefort

**Affiliations:** 1 Plants and Pathogens GroupResearch Institute Land Nature Landscape, HEPIA Geneva School of Engineering, Architecture and Landscape, HES-SO University of Applied Sciences and Arts Western Switzerland, 150 Route de Presinge, Jussy, 1254 Switzerland; 2 Laboratory of Microbiology, Food Technology and Phytopathology, Faculty of Science and Technology, University of Abomey-Calavi, 05 P.O. Box 1604, Abomey-Calavi, Benin; 3 Laboratory of Biology and Molecular Typing in Microbiology, Faculty of Science and Technology, University of Abomey-Calavi, 04 P.O. Box 1107, Cotonou, Benin

**Keywords:** Abiotic stress, PGPB, Crop yield enhancement, Biopriming, Bioinoculants, Sustainable agriculture

## Abstract

High soil salinity constrains global agricultural productivity, necessitating effective mitigation strategies. Plant growth-promoting bacteria (PGPB) offer a promising approach to enhance crop salt tolerance. This study evaluated halotolerant bacteria from *O. dillenii* to improve tomato (*Solanum lycopersicum* L.) growth under salinity. Bacteria from plant organs and rhizosphere were screened at 0–3 M NaCl; 52.38% grew above 1.5 M, and six strains—*P. flexa* S4, S6, R7, C5; *B. amyloliquefaciens* R4; and *M. yunnanensis* C11—tolerated up to 2.5 M. Under 120 mM NaCl, biopriming with *P. flexa* S6 and C5 increased germination to 75% and vigor index by 149.2%. Under 200 mM NaCl, *P. flexa* S6 boosted root biomass by 167.5%, C5 improved root length and diameter by 25.6% and 30.98%, and R7 enhanced shoot growth while reducing leaf proline by 78%. These selected strains effectively alleviate salt stress and promote tomato growth, promising an application in sustainable agricultural systems.

**Graphical abstract:**

**Figure:**
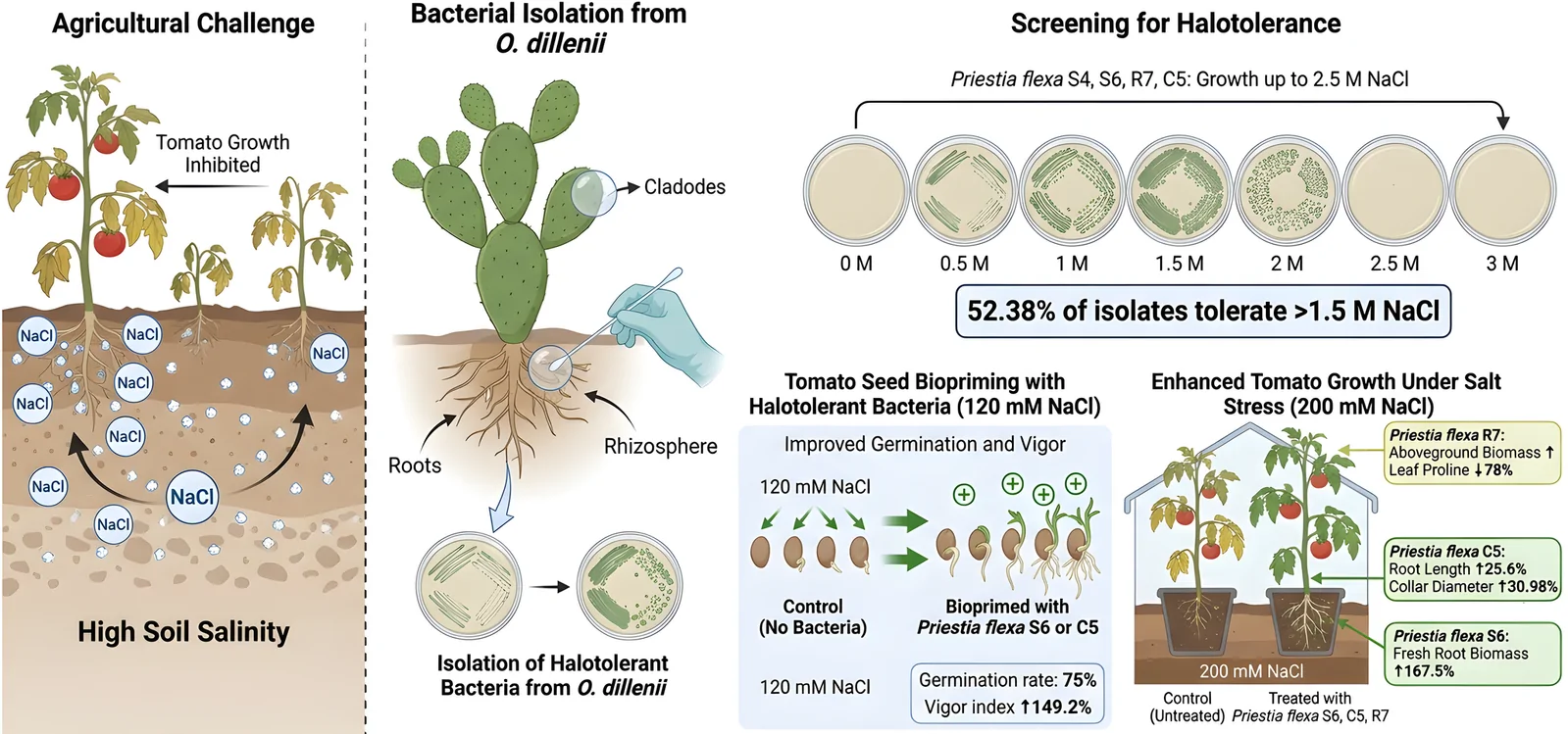

## Introduction

The continuous increase in human population and human activities causing climate change are major challenges facing global food security (Rafique et al. 2025). Globally, more than 900 million hectares of land, including about one-third of irrigated agricultural land, are affected by saline-induced stress (Zhou et al. 2024). Soil salinity affects plant growth and soil conditions through several interrelated physiological mechanisms, including osmotic stress, ionic toxicity, and oxidative stress. High salt concentrations in the soil reduce soil water potential, thereby limiting water uptake by the plant roots and leading to reduced growth. Excessive accumulation of sodium (Na⁺) and chloride (Cl⁻) ions disrupts nutritional balance, enzymatic activity, and membrane integrity in plants. Furthermore, salinity promotes the excessive production of reactive oxygen species (ROS), which cause cellular damage affecting proteins, lipids, and nucleic acids. These disruptions impair photosynthesis, metabolism, and plant development, ultimately leading to reduced agricultural productivity (Abbas and Al-Naemi 2026; Chen et al. 2026).

The tomato (*Solanum lycopersicum* L.) is one of the world’s most important vegetable crops with high nutritional, economical, and industrial value. It is rich in vitamins, minerals, and antioxidants, which plays a central role in human nutrition and as a raw material to the food processing industry (Huang et al. 2026). In 2024, global tomato production was estimated at approximately 188 million metric tons, while Benin’s production reached nearly 288,000 metric tons, representing a 2.9% increase over the previous year (FAOSTAT 2026). Despite this growth, tomato cultivation continues to face several abiotic constraints that limit its production potential.

In Benin, coastal areas are among the most vulnerable regions affected by climate change (Bonou et al. 2024). The sudden rising in the sea levels can facilitate the intrusion of seawater into aquifers and coastal farmland, contributing to increased salinity in soils and water resources (Agossou et al. 2022). This situation represents a growing threat to coastal vegetable production systems, where tomato cultivation is particularly affected by salinity stress.

Furthermore, the intensification of agricultural practices aimed at meeting growing food demand has been linked to the use of chemical fertilizers and pesticides. However, only 20 to 30% of the applied fertilizers are actually taken up by plants (Saeed et al. 2021). Excessive use of these inputs, mostly influence the physicochemical and biological properties of soils, alter the structure of microbial communities, and contribute to the decline in soil fertility. In this context, developing sustainable agricultural strategies to enhance crop productivity while minimizing environmental impacts became imperative.

Plant growth-promoting bacteria (PGPB) constitute a diverse group of beneficial microorganisms associated with the plant rhizosphere, endosphere, and phyllosphere, where they establish synergistic interactions with their host plants (Nabti et al. 2026). The most frequently reported genera include *Bacillus*, *Pseudomonas*,* Azoarcus*,* Azospirillum*,* Azotobacter*,* Enterobacter*,* Serratia*,* Streptomyces*,* Herbaspirillum*,* Klebsiella*,* Alcaligenes*,* Arthrobacter*,* Burkholderia*,* Microbacterium*,* Micrococcus*,* Pantoea*, and *Stenotrophomonas* (Noumavo et al. 2015; Ahmad et al. 2017; Krause et al. 2017; Tavares et al. 2018; Ludueña et al. 2019; Passari et al. 2019; Andreozzi et al. 2019; Saranraj et al. 2023; Mametja et al. 2025). Beyond the effect of beneficial microorganisms on plant growth, PGPB helps mitigate salt stress by regulating osmotic balance, stimulating antioxidant defense mechanisms, and improving nutrient availability and absorption. Siderophore production, phosphate solubilization, and ACC deaminase activity are among the key mechanisms involved in these responses (Fouad et al. 2026).

Cacti (Cactaceae) exhibit remarkable adaptations that enable them to survive and grow under harsh environmental conditions, including water scarcity, high salinity, and nutrient limitation. These conditions favor the establishment of salinity tolerant microbial communities with high biotechnological potential. Among them, *O. dillenii* (Ker Gawl.) Haw is one of the dominant plant species in the coastal zone of Benin. Over the past decade, there has been growing interest in exploring the microbiomes associated with these plants to maximize their potential and minimize the ecological constraints associated with their cultivation. Current evidence suggests that these plants actively recruit beneficial microorganisms that contribute to their adaptation to adverse environmental conditions (Qadir et al. 2024). The saline habitat of *O. dillenii* suggests that its associated bacteria may possess adaptive traits that could support tolerance to environmental stressors. Brun et al. (2026) recently characterized bacteria isolated from cladode, roots and rhizosphere of *O. dillenii*, which harboured plant growth promoting traits.

The present study was designed to evaluate the halotolerant potential of these PGPB to improve growth and salt stress tolerance in tomato.

## Materials and methods

### Bacterial strains

The bacterial strains used in this study were isolated from the endosphere and rhizosphere of *O. dillenii* collected in the coastal region of Benin. A total of 31 bacterial strains previously reported by Brun et al. (2026) were initially available. Strains identified as known or potential human pathogens, opportunistic pathogens, or members of bacterial groups associated with biosafety concerns were not considered for the present study. Finally, 21 isolates were retained for the present study (Table 1). All isolates were preserved at − 20 °C in Luria–Bertani broth (LBB; Roth AG, Arlesheim, Switzerland) supplemented with 30% glycerol until use.

### In vitro screening of halotolerant bacterial strains

The sodium chloride (NaCl) tolerance of bacterial strains was evaluated on Luria–Bertani (LBA; Roth AG) supplemented with different concentrations of NaCl: 0% (0 M), 0.29% (0.05 M), 0.58% (0.1 M), 1.17% (0.2 M), 2.34% (0.4 M), 3.51% (0.6 M), 4.68% (0.8 M), 5.84% (1 M), 8.77% (1.5 M), 11.69% (2 M), 14.61% (2.5 M), and 17.53% (3 M), according to the method described by Teker Yıldız and Acar (2025). The bacterial strains were streaked from pure cultures and then incubated at 30 °C for 48 h. Each treatment was performed in triplicate. Bacterial growth was assessed relative to the NaCl-free control and classified into three categories: abundant growth (* ++*), moderate growth (* +*), or no growth (* -*).

### Effect of bacterial biopriming on tomato seed germination and early seedling growth under in vitro salt stress

The best ten (10) bacterial strains, selected for salt tolerance, were cultured separately in 30 mL of Luria–Bertani broth (LBB; Roth AG) for 24 h at 30 °C under constant agitation (125 rpm). The bacterial cultures were then centrifuged at 8,000 rpm for 5 min, and the pelletized cells were resuspended in sterile 1X phosphate-buffered saline (PBS; Roth AG). The concentration of the bacterial suspensions was standardized to approximately 10⁸ CFU mL⁻¹ based on measurements of optical density at 600 nm (OD₆₀₀ = 0.8–1.0). Tomato seeds of the cultivar Rougella RZ F1 (Rijk Zwaan, De Lier, The Netherlands) (*N* = 660) were surface sterilized by immersion in 70% ethanol for 2 min, followed by treatment with 1% sodium hypochlorite for 3 min (Sharma et al. 2021).

Then, the seeds were rinsed three times with sterile demineralized water and then dried on sterile Whatman filter paper (Rotilabo^®^, Germany). For each treatment, 30 seeds were used, divided into three replicates of 10 seeds each. Biopriming was performed by immersing the seeds in 15 mL of bacterial suspension for 30 min (Singh et al. 2024).

The experiment was conducted under two experimental conditions: (i) a control condition without salt stress, in which the filter papers were moistened with sterile demineralized water, and (ii) a salt-stress condition achieved by applying a 120 mM NaCl solution, a concentration previously determined to be the minimum dose capable of reducing the germination rate by approximately 50%. The treated seeds were placed on the surface of sterile, pre-moistened filter papers, which were then placed in sealed Petri dishes and incubated at 23 ± 1 °C in the dark.

After 10 days of incubation, the germination percentage was determined by considering seeds with visible emergence of the radicle as germinated. At the end of the incubation, seedling growth parameters were evaluated. The lengths of the radicle and hypocotyl were measured using a digital caliper (Profi Scale^®^, Germany) on fifteen seedlings per treatment (five seedlings per replicate). Only seedlings with a root length greater than 1 mm were included in the growth measurements. The Seedling Vigor Index (SVI-I) was calculated according to the method of Abdul-Baki and Anderson (1973) using the following formula:

$$\:\mathrm{SVI-I}=\mathrm{G}\mathrm{e}\mathrm{r}\mathrm{m}\mathrm{i}\mathrm{n}\mathrm{a}\mathrm{t}\mathrm{i}\mathrm{o}\mathrm{n}\:\mathrm{p}\mathrm{e}\mathrm{r}\mathrm{c}\mathrm{e}\mathrm{n}\mathrm{t}\mathrm{a}\mathrm{g}\mathrm{e}\mathrm{\:(\%)}\times\:\mathrm{A}\mathrm{v}\mathrm{e}\mathrm{r}\mathrm{a}\mathrm{g}\mathrm{e}\:\mathrm{s}\mathrm{e}\mathrm{e}\mathrm{d}\mathrm{l}\mathrm{i}\mathrm{n}\mathrm{g}\:\mathrm{l}\mathrm{e}\mathrm{n}\mathrm{g}\mathrm{t}\mathrm{h}\:\mathrm{(cm)}$$

where the average seedling length is the sum of the average radicle length and the average hypocotyl length.

### Evaluation of the effect of bacterial strains on tomato growth under in-planta salinity stress

#### Experimental conditions

The *in-planta* experiment was conducted from April 29 to June 10, 2026, in a Venlo-type greenhouse (9.60 m × 9.00 m; eave height: 5.80 m; ridge height: 6.70 m; roof slope: 22°; 4-mm glass covering). Daytime temperature was maintained at 24 °C, with ventilation activated at 26 °C, while nighttime temperature was maintained at 18 °C. Relative humidity was maintained at approximately 60%, and no artificial lighting was used. During the experimental period, the mean recorded temperature was 22.7 °C and the mean relative humidity was 43%.

#### Experimental design

Of the ten bacterial strains preselected during the salt-stress germination assays, the five most effective strains, i.e. halotolerant and beneficial to tomato germination under salt stress, were selected for greenhouse evaluation. A total of 120 pots (2 L) were filled with the horticultural substrate Klasmann Substrat 1 (Eric Schweizer AG, Thun, Switzerland), consisting of a moderately fertilized potting mixture (0.8 kg m⁻³ NPK 14–10–18; pH 6.0 ± 0.3). Prior to use, the substrate was sterilized by heat treatment for 2 h using a Harter Sterilo 1 K soil sterilizer (Jarditech, Clarens, Switzerland). The pots were subsequently filled following a standardized procedure consisting of dropping each pot three times from a height of approximately 10 cm onto a rigid surface to ensure uniform substrate compaction. A 15-cm-diameter saucer was placed beneath each pot. Tomato seeds were sown directly into the pots at a depth of approximately 6 mm, with one seed per pot. The experiment was arranged in a completely randomized design comprising 12 treatments. Under non-saline conditions, the five selected bacterial strains and an uninoculated control were irrigated with tap water. The same treatments were evaluated under saline conditions. Each treatment consisted of ten replicates, resulting in a total of 120 experimental units (Arminjon and Lefort 2025).

#### Bacterial inoculation and application of saline stress

Fourteen (14) days after sowing, the seedlings were inoculated at the collar with 5 mL of a bacterial suspension adjusted to approximately 10⁸ CFU mL⁻¹. Forty-eight (48) hours after inoculation, salinity stress was gradually imposed on the designated treatment groups to avoid osmotic shock. A NaCl solution was applied at a rate of 200 mL per pot until final concentrations of 50, 100, 150, and 200 mM were reached after 2, 4, 6, and 8 days, respectively (Abdelshafy Mohamad et al. 2020). The final concentration of 200 mM NaCl was selected to impose a severe but non-lethal salinity stress, allowing a clear evaluation of bacterial strain effectiveness under highly restrictive growth conditions. This concentration has been previously used in tomato salinity studies and induces substantial growth reduction while maintaining plant survival (Arminjon and Lefort 2025). Plants grown under non-saline conditions received the same volume of tap water according to the same irrigation schedule. Twenty-eight (28) days after sowing, the plants were supported with wooden stakes to maintain an upright growth habit.

#### Evaluation of growth parameters

After six (6) weeks of cultivation under the respective treatment conditions, all plants were evaluated. Plant height was measured from the collar to the tip of the longest leaf using a folding rule. Root length was determined from the collar to the tip of the primary root after carefully removing the plants from the pots and washing the root system. Stem diameter was measured at the collar using an electronic caliper (Profi Scale^®^, Germany). The number of leaves was determined by direct counting. Fresh weights of the shoots and roots were recorded immediately after harvest using an analytical balance (Mettler Toledo^®^, Greifensee, Switzerland). The samples were subsequently dried in an oven at 45 °C for 96 h until constant weight was achieved, and the corresponding dry weights were then determined.

#### Determination of proline content in leaves

The proline content was determined using the ninhydrin colorimetric method described by Carillo et al. (2011). For each sample, 50 mg of fresh leaf tissue was homogenized in 1 mL of a mixture ethanol: water (40:60, v/v). The extracts were then incubated at 4 °C for 24 h before centrifugation at 14,000 g for 5 min. Proline quantification was performed using a ninhydrin reagent prepared in a solution containing 1% (m/v) ninhydrin, 60% (v/v) acetic acid, and 20% (v/v) ethanol. In 1.5 mL Eppendorf Safe-Lock^®^ tubes (Roth AG) 1,000 µL of ninhydrin reagent was mixed with 500 µL plant extract. A standard curve was established using L-proline solutions (0.2, 0.5, 1, 2, and 5 mM) prepared in the same extraction medium. The reaction mixtures were homogenized and then incubated at 95 °C for 20 min. After cooling, the samples were centrifuged at 10,000 rpm for 1 min. The resulting supernatant was transferred to a spectrophotometric cuvette, and the absorbance was measured at 520 nm using a Biomate 160 UV–visible spectrophotometer (Thermo Fisher Scientific). The proline concentration was determined by comparison with the calibration curve and expressed as µmol g⁻¹ fresh weight.

### Statistical analyses

All statistical analyses were performed using R software version 4.6.1 (R Core Team, 2026). The data were subjected to an analysis of variance (ANOVA). In cases of a significant effect (*p* < 0.05), means were compared using Tukey’s test at the 5% significance level. The results are presented as means ± standard error. A heatmap was generated using the ggplot2 package in R to visualize the growth responses of bacterial strains under increasing NaCl concentrations.

**Table 1:** Origin, taxonomic identification, plant growth-promoting traits of 21 bacterial strains isolated from cladodes, roots, and rhizosphere of *O. dillenii*

Origin/source	Codes	GenBank accession no.	Identity	Functional characteristics (Brun et al. 2026)
Cladode	C1	PZ149653	*Priestia flexa*	Nitrogen fixation, phosphate solubilization, protease, and amylase activity
	C2	PZ149654	*Priestia flexa*	Nitrogen fixation, phosphate solubilization, protease, and amylase activity
	C5	PZ149656	*Priestia flexa*	EPS production, nitrogen fixation, phosphate solubilization, IAA production, protease, and lipase activity
	C6	PZ149657	*Priestia flexa*	EPS production, nitrogen fixation, IAA production, protease, and lipase activity
	C7	PZ149658	*Bacillus subtilis*	EPS production, nitrogen fixation, siderophore production, phosphate solubilization, IAA production, and protease activity
	C8	PZ149659	*Bacillus amyloliquefaciens*	EPS production, nitrogen fixation, siderophore production, phosphate solubilization, IAA production, protease, and lipase activity
	C10	PZ149660	*Priestia flexa*	Nitrogen fixation, siderophore production, phosphate solubilization, IAA production, and protease activity
	C11	PZ102205	*Micrococcus yunnanensis*	EPS production, nitrogen fixation, siderophore production, phosphate solubilization, IAA production, and amylase activity
	C12	PZ149661	*Bacillus tropicus*	Phosphate solubilization, IAA production, and protease activity
Root	R3	PZ149663	*Bacillus subtilis*	EPS production, nitrogen fixation, siderophore production, phosphate solubilization, protease, and lipase activity
	R4	PZ149664	*Bacillus amyloliquefaciens*	EPS production, nitrogen fixation, siderophore production, phosphate solubilization, protease, and lipase activity
	R6	PZ149665	*Bacillus subtilis*	EPS production, nitrogen fixation, siderophore production, phosphate solubilization, protease, lipase, and amylase activity
	R7	PZ149666	*Priestia flexa*	Nitrogen fixation, siderophore production, phosphate solubilization, protease, and amylase activity
	R10	PZ102216	*Pseudochrobactrum asaccharolyticum*	Siderophore production and phosphate solubilization
Soil	S1	PZ102218	*Microbacterium aborescens*	Siderophore production, phosphate solubilization, IAA production, and protease activity
	S2	PZ102219	*Heyndrickxia oleronia*	Siderophore production, phosphate solubilization, and lipase activity
	S4	PZ149669	*Priestia flexa*	Nitrogen fixation, phosphate solubilization, and protease activity
	S5	PZ149670	*Priestia flexa*	Nitrogen fixation, phosphate solubilization, protease, and amylase activity
	S6	PZ149671	*Priestia flexa*	Nitrogen fixation, phosphate solubilization, protease, and amylase activity
	S7	PZ149672	*Bacillus subtilis*	EPS production, nitrogen fixation, siderophore production, phosphate solubilization, protease, and lipase activity
	S8	PZ149673	*Priestia flexa*	Nitrogen fixation, siderophore production, phosphate solubilization, protease, and amylase activity

*EPS* exopolysaccharide production; *IAA* indole-3-acetic acid production

## Results

### NaCl tolerance of bacterial strains

**Fig. 1:**
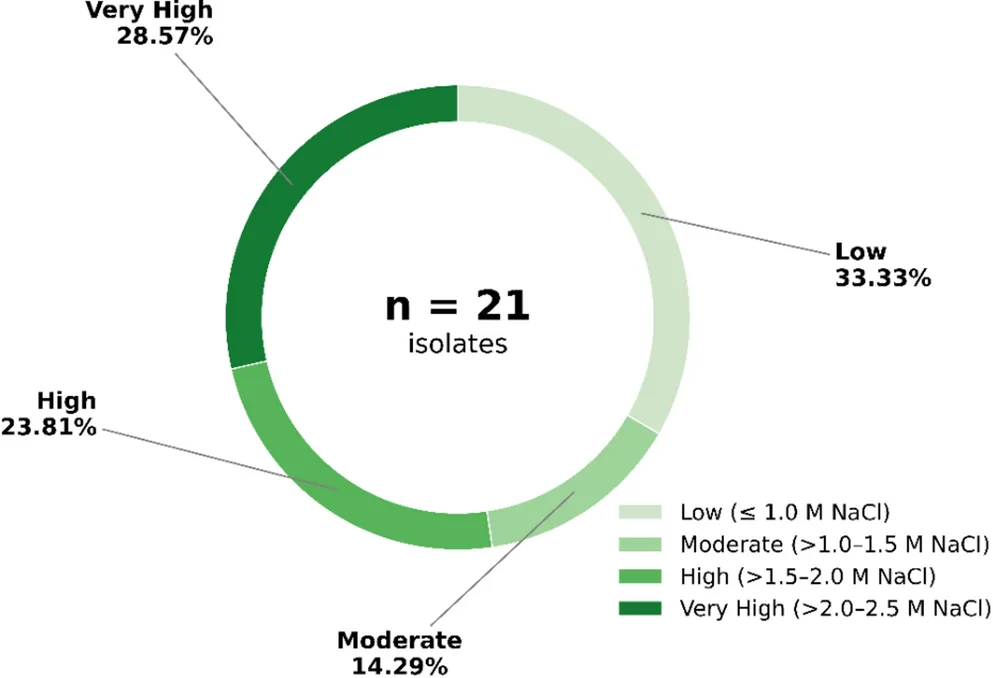
Distribution of bacterial strains according to their level of halotolerance (low, moderate, high, very high)

**Fig. 2:**
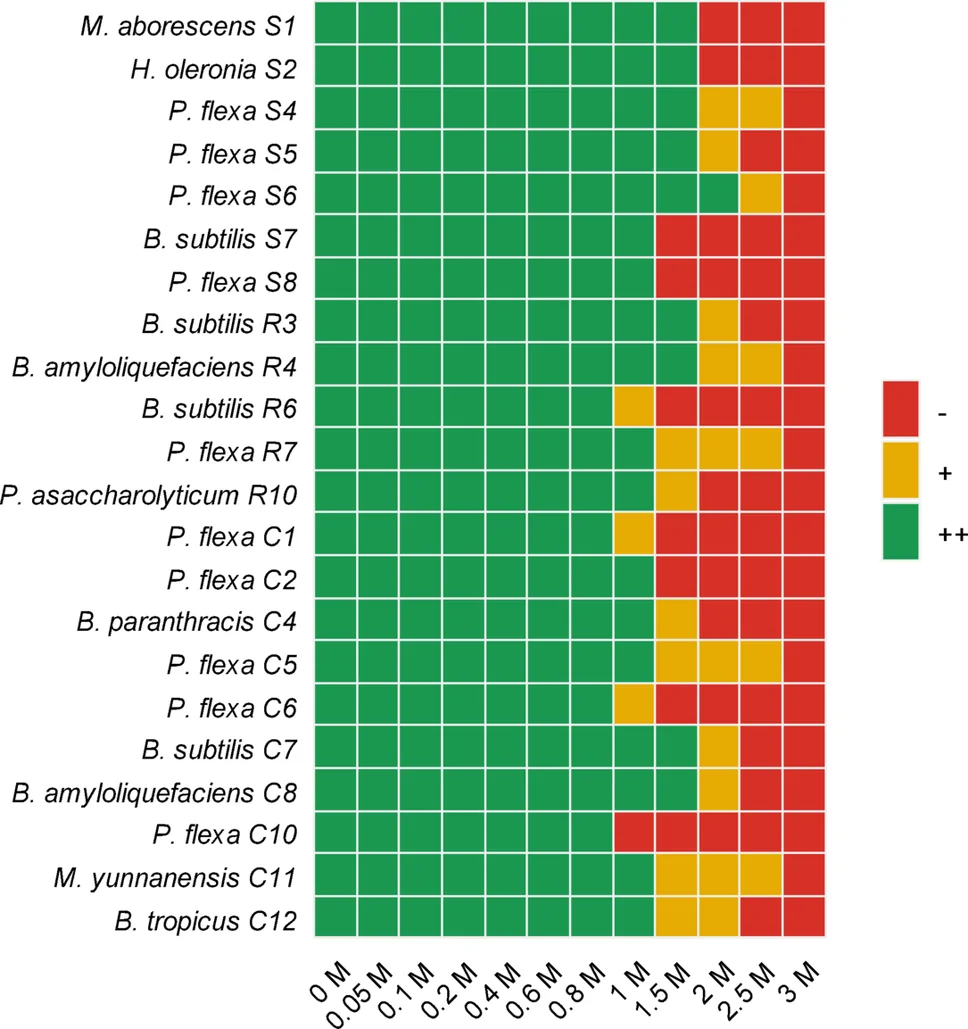
Heatmap of bacterial growth responses under increasing NaCl concentrations (0–3 M). Key: abundant growth (* ++*), moderate growth (* +*), no growth (* -*)

**Fig. 3:**
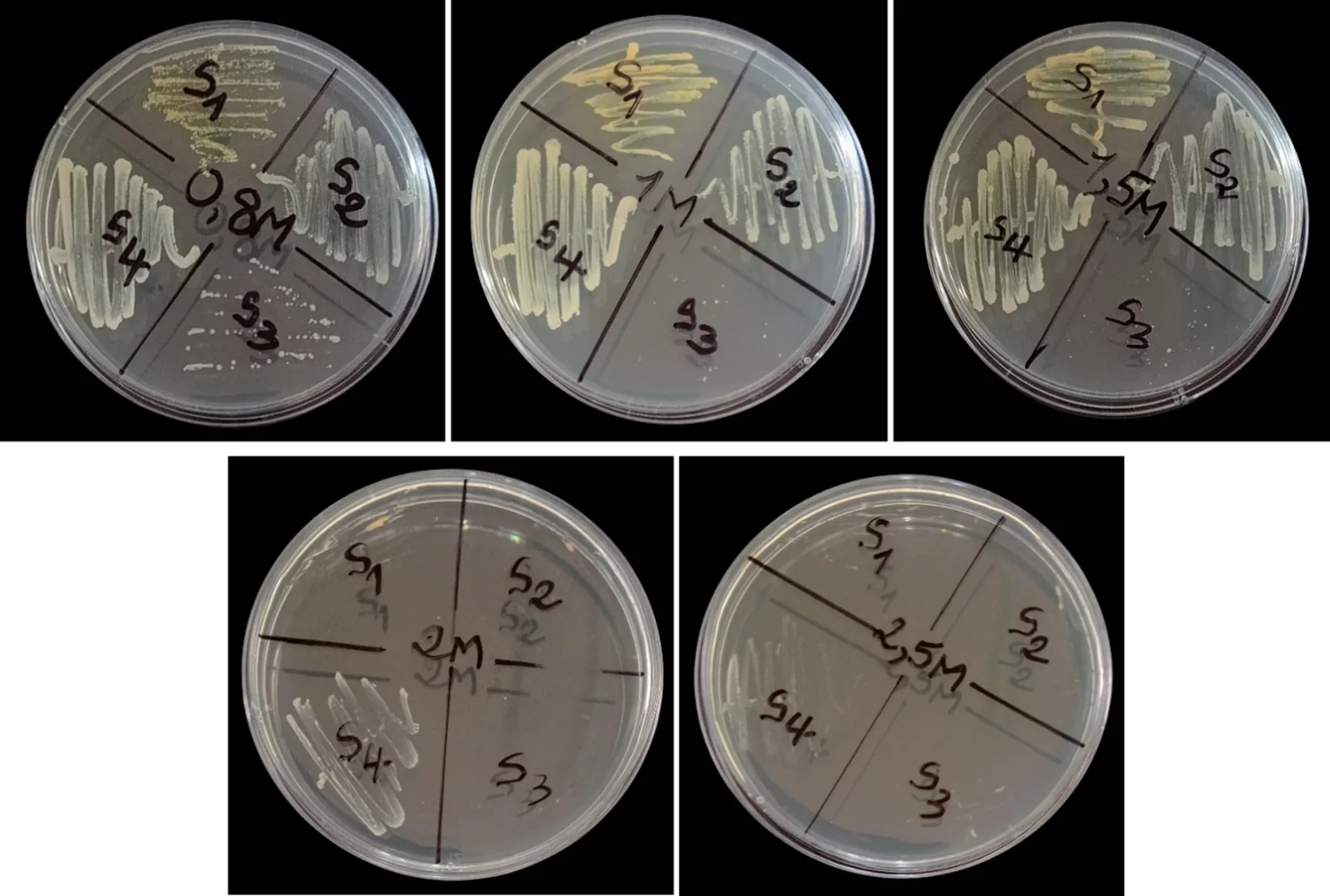
Growth responses of bacterial strains on LBA supplemented with increasing NaCl concentrations (0.8–2.5 M) after 48 h of incubation

The halotolerance in vitro screening revealed a progressive decline in the number of strains capable of growing as the NaCl concentration increased, with no growth observed at 3 M for the 21 bacterial strains; 33.33% exhibited low halotolerance (≤ 1.0 M NaCl), while 14.29% were moderately halotolerant (> 1.0–1.5 M). In contrast, 52.38% of the bacterial strains exhibited high to very high halotolerance, maintaining growth at NaCl concentrations above 1.5 M. Among these, six bacterial strains, namely, *P. flexa* S4, *P. flexa* S6, *B. amyloliquefaciens* R4, *P. flexa* R7, *P. flexa* C5, and *M. yunnanensis* C11 exhibited growth up to 2.5 M NaCl, corresponding to the maximum tolerance level observed. Considering all bacterial strains capable of growing at concentrations ≥ 2.0 M, the ten most halotolerant strains included *P. flexa* S5, *B. subtilis* R3, *B. subtilis* C7, and *B. amyloliquefaciens* C8 (Figs. 1, 2 and 3).

### Effect of bacterial biopriming on the germination rate and vigor index of tomato seeds under saline and non-saline conditions

**Fig. 4:**
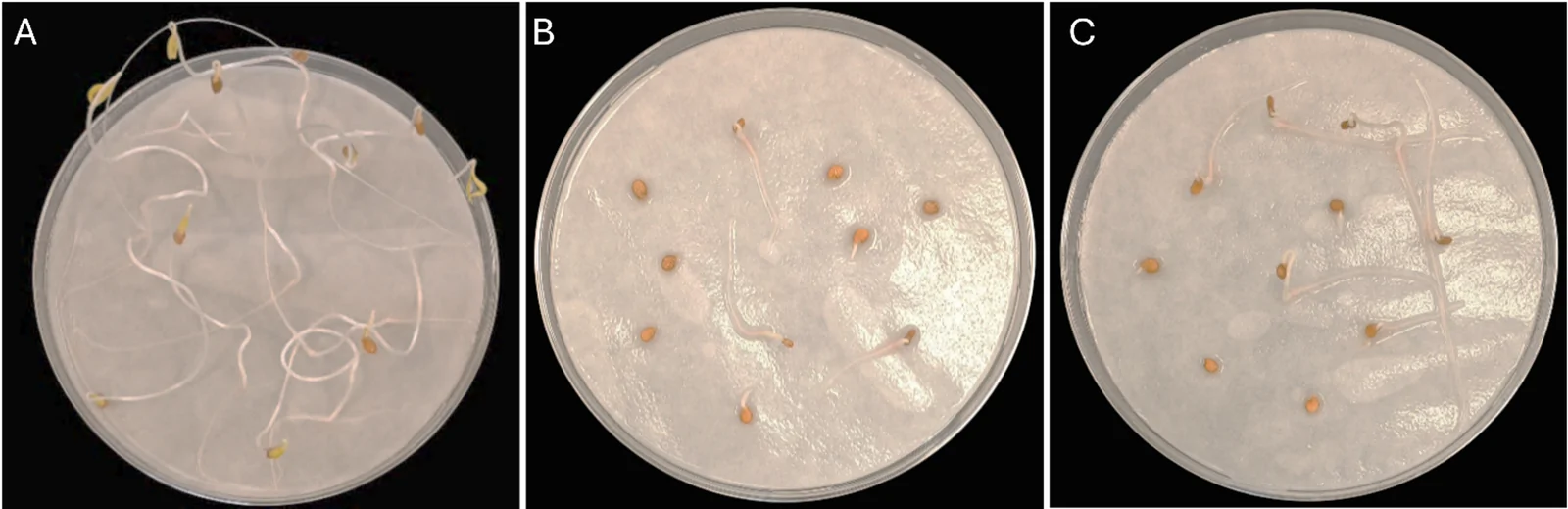
Effect of bacterial strains on tomato seed germination after 10 days of incubation. **A** No-salt control (0 mM), **B** salt control (120 mM), **C** *P. flexa* S6 + 120 mM

**Fig. 5:**
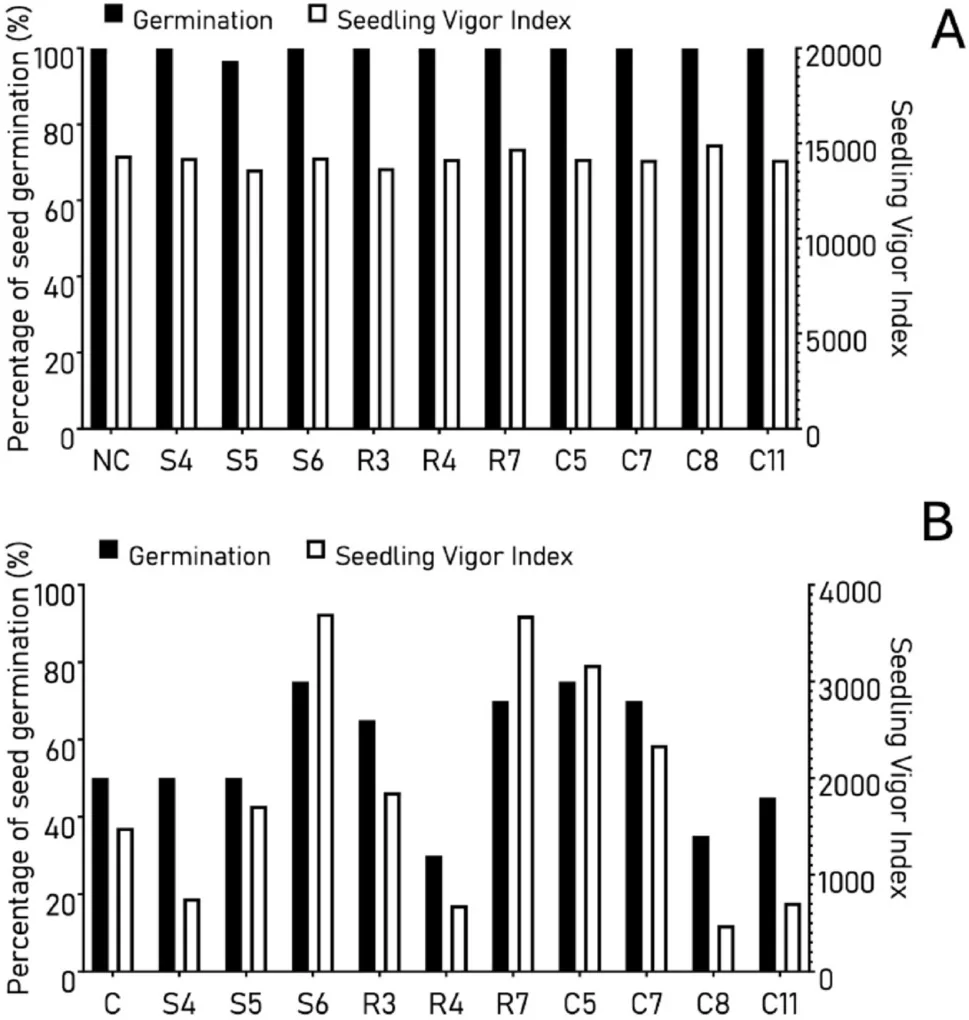
Effect of bacterial biopriming on the germination rate and vigor index of tomato seeds in the absence of salt stress (**A**) and under salt stress (**B**). NC: no-salt control, C: salt control; S4: *P. flexa* S4; S5: *P. flexa* S5; S6: *P. flexa* S6; R3: *B. subtilis* R3; R4: *B. amyloliquefaciens* R4; R7: *Priestia flexa* R7; C5: *P. flexa* C5; C7: *B. subtilis* C7; C8: *B. amyloliquefaciens* C8; C11: *M. yunnanensis* C11

In the absence of salt stress, all strains maintained a germination rate approximately 100%. The highest vigor indices were recorded for *B. amyloliquefaciens* C8 (+ 4.0%) and *P. flexa* R7 (+ 2.5%) compared to the non-inoculated control. Under 120 mM NaCl, salinity reduced the germination rate of the control seeds to 50% and decreased the vigor index to 1,485.5. Seed biopriming improved both parameters. *P. flexa* S6 and *P. flexa* C5 increased the germination rate to 75%. The greatest increases in vigor index were observed in seeds inoculated with *P. flexa* S6 (+ 149.2%), *P. flexa* R7 (+ 147.9%), and *P. flexa* C5 (+ 113.6%) compared to the saline control (Figs. 4 and 5).

### Effect of bacterial strains on hypocotyl and radicle growth in 10-day-old tomato seedlings

The results revealed no significant differences among treatments for hypocotyl length (F = 0.321, *p* = 0.9722) or radicle length (F = 0.766, *p* = 0.6604). Nevertheless, the greatest increases in hypocotyl length compared to the no-salt control were observed with *B. amyloliquefaciens* C8 (+ 7.3%), followed by *B. subtilis* C7 (+ 5.8%), *M. yunnanensis* C11 (+ 5.0%), and *P. flexa* C5 (+ 4.4%). For radicle length, the highest increase was observed with *M. yunnanensis* C11 (+ 6.5%), followed by *P. flexa* R7 (+ 3.9%) and *B. amyloliquefaciens* C8 (+ 1.2%) (Table 2).

**Table 2:** Effect of bacterial strains on hypocotyl and radicle lengths in 10-day-old tomato seedlings

Treatment	Hypocotyl length (mm)	Radicle length (mm)
No-salt control	66.54 ± 3.21^a^	77.15 ± 2.73^a^
*P. flexa* S4	66.79 ± 3.08^a^	75.50 ± 4.36^a^
*P. flexa* S5	69.13 ± 4.54^a^	71.98 ± 4.25^a^
*P. flexa* S6	67.55 ± 4.28^a^	74.95 ± 3.95^a^
*B. subtilis* R3	64.91 ± 4.13^a^	72.17 ± 3.13^a^
*B. amyloliquefaciens* R4	67.12 ± 4.06^a^	74.78 ± 3.79^a^
*P. flexa* R7	67.09 ± 3.20^a^	80.14 ± 3.92^a^
*P. flexa* C5	69.46 ± 2.97^a^	72.50 ± 3.08^a^
*B. subtilis* C7	70.37 ± 1.93^a^	71.05 ± 5.10^a^
*B. amyloliquefaciens* C8	71.40 ± 2.67^a^	78.09 ± 4.35^a^
*M. yunnanensis* C11	69.86 ± 2.77^a^	82.14 ± 6.18^a^
P-value	0.9722	0.6604
F	0.321	0.766

Within the same column, means marked with different letters are significantly different at the 5% level according to the Student-Newman-Keuls test

### Effect of bacterial strains on hypocotyl and radicle growth in 10-day-old tomato seedlings under salt stress

**Fig. 6:**
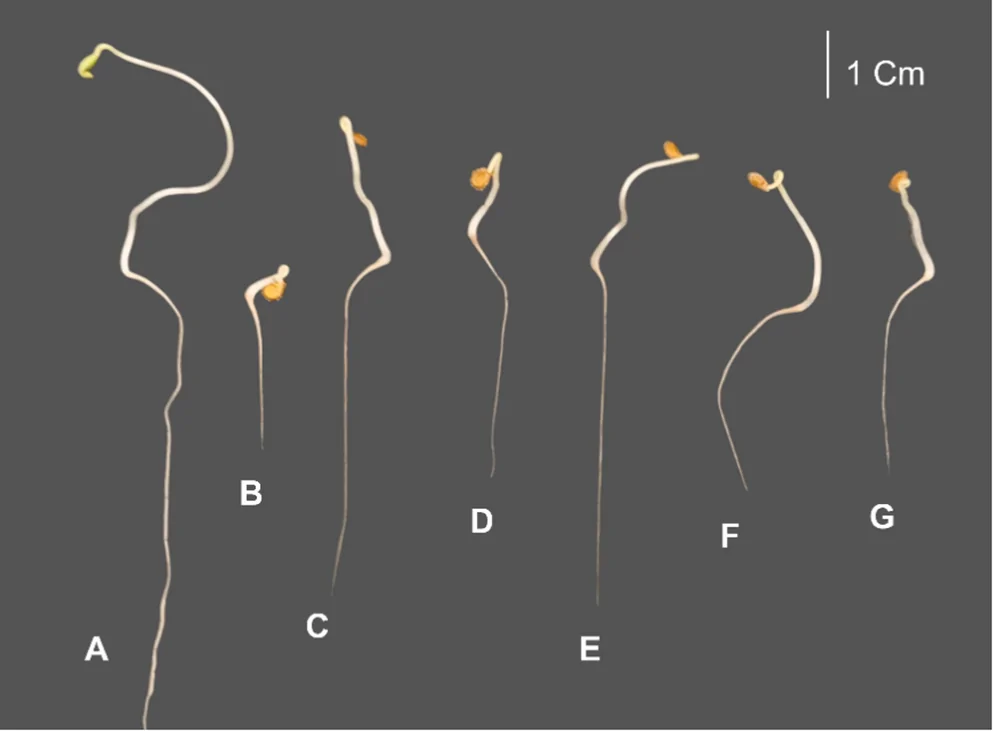
10-day-old tomato seedlings treated with bacterial isolates. A: no-salt control (0 mM), B: salt control (120 mM), C: *P. flexa* R7 + 120 mM, D: *P. flexa* C5 + 120 mM, E: *P. flexa* S6 + 120 mM, F: *B. subtilis* C7 + 120 mM, G: *B. subtilis* R3 + 120 mM

The results revealed a significant effect of treatments on hypocotyl length (F = 3.91, *p* = 0.00047) and radicle length (F = 7.04, *p* < 0.0001). For hypocotyl length, the greatest increases relative to the salt control were observed with *P. flexa* R7 (+ 110%), followed by *P. flexa* C5 (+ 62%) and *P. flexa* S6 (+ 61%). For radicle length, the greatest increases were observed with *P. flexa* S6 (+ 68%) and *P. flexa* R7 (+ 67%), followed by *P. flexa* C5 (+ 36%) (Fig. 6; Table 3).

**Table 3:** Effect of bacterial strains on hypocotyl and radicle lengths of 10-day-old tomato seedlings under salt stress

Treatment	Hypocotyl length (mm)	Radicle length (mm)
Salt control	6.92 ± 1.34^b^	22.79 ± 3.98^b^
*P. flexa* S4	4.10 ± 0.35^b^	11.04 ± 2.19^b^
*P. flexa* S5	6.81 ± 1.26^b^	27.45 ± 4.71^a^
*P. flexa* S6	11.16 ± 2.78^a^	38.21 ± 5.20^a^
*B. subtilis* R3	6.95 ± 2.20^b^	21.56 ± 4.90^b^
*B. amyloliquefaciens* R4	4.78 ± 0.63^b^	18.08 ± 2.55^b^
*P. flexa* R7	14.53 ± 0.99^a^	38.09 ± 2.82^a^
*P. flexa* C5	11.20 ± 2.40^a^	31.10 ± 5.71^a^
*B. subtilis* C7	9.59 ± 2.99^a^	23.89 ± 2.79^a^
*B. amyloliquefaciens* C8	3.72 ± 0.33^b^	9.98 ± 1.85^b^
*M. yunnanensis* C11	5.01 ± 1.34^b^	10.85 ± 3.01^b^
P-value	0.00047	< 0.0001
F	3.91	7.04

Within the same column, means marked with different letters are significantly different at the 5% level according to the Student-Newman-Keuls test

### Effect of bacterial inoculation on the height of tomato seedlings under salt stress

**Fig. 7:**
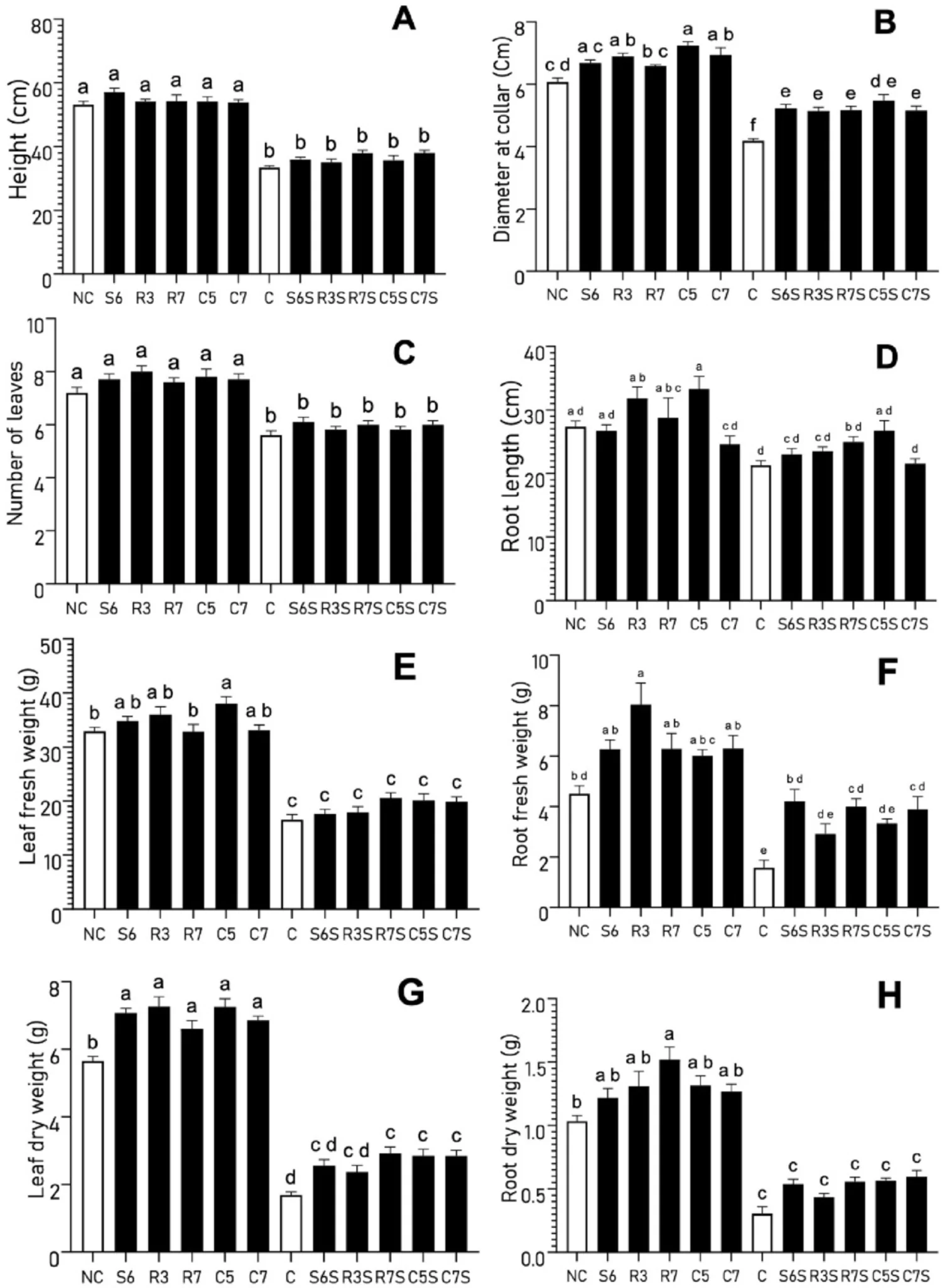
**A**–**H** Effects of bacterial strains on growth parameters of inoculated and uninoculated tomato seedlings, in the absence and presence of 200 mM NaCl (*n* = 10). Values followed by the same letter are not significantly different (*p* > 0.05). NC: no-salt control; S6: *P. flexa* S6; R3: *B. subtilis* R3; R7: *Priestia flexa* R7; C5: *P. flexa* C5; C7: *B. subtilis* C7; C: salt control (200 mM NaCl); S6S: *P. flexa* S6 + 200 mM NaCl; R3S: *B. subtilis* R3 + 200 mM NaCl; R7S: *Priestia flexa* R7 + 200 mM NaCl; C5S: *P. flexa* C5 + 200 mM NaCl; C7S: *B. subtilis* C7 + 200 mM NaCl

**Fig. 8:**
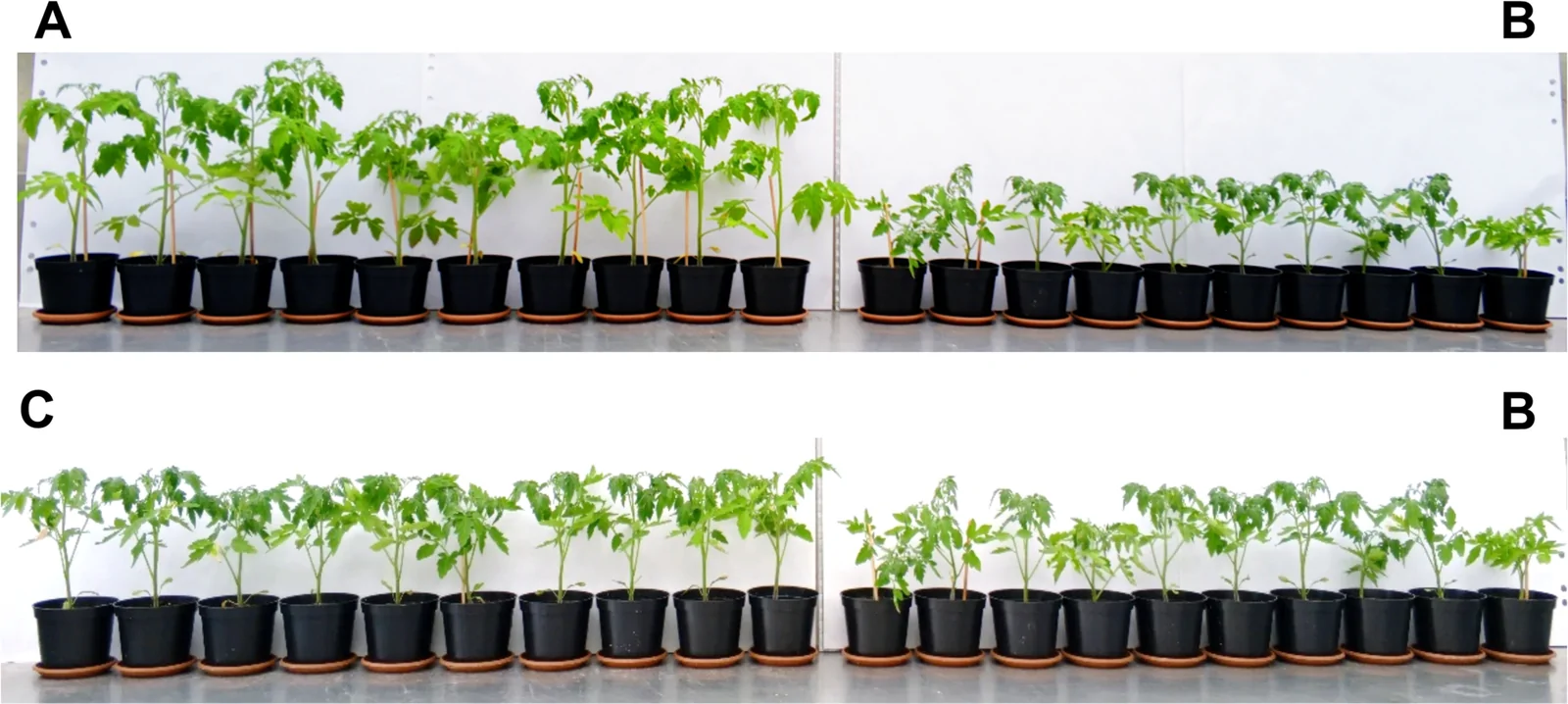
Effects of salt stress and inoculation with *B. subtilis* C7 on tomato seedlings after six weeks of growth: **A** no-salt control, **B** salt control (200 mM NaCl), and **C** seedlings inoculated with *B. subtilis* C7 under 200 mM NaCl

The effect of bacterial inoculation on the growth parameters of tomato seedlings is presented in Fig. 7A–H. The results showed revealed a highly significant effect of treatments on tomato seedling height [F (11,108) = 68.38, *p* < 0.0001]. However, multiple comparisons indicated no significant differences among treatments within the same salinity level. Under non-saline conditions, the tomato seedlings inoculated with *P. flexa* S6 showed plant length of 57.05 cm, corresponding to a 7.46% increase compared to the uninoculated control. Exposure to 200 mM NaCl reduced the height of control seedlings by 37.09%. Under saline conditions, the highest plant heights were observed in seedlings inoculated with *B. subtilis* C7 and *P. flexa* R7, which reached mean heights of 37.94 and 37.90 cm, respectively. These values corresponded to increases of 13.59% and 13.47% relative to the salt control (Figs. 7A and 8).

### Effect of bacterial inoculation on the collar diameter of tomato seedlings under salt stress

The results revealed a highly significant effect of treatments on collar diameter [F (11,108) = 52.06, *p* < 0.0001]. Under non-saline conditions, collar diameter ranged from 6.07 to 7.24 mm. *P. flexa* C5 exhibited the highest mean collar diameter (7.24 mm), corresponding to a 19.26% increase compared to the uninoculated control (6.07 mm). *B. subtilis* C7 (14.26%), *B. subtilis* R3 (13.61%), *P. flexa* S6 (10.09%), and *P. flexa* R7 (8.40%) also showed an increase in the collar diameter. Exposure of tomato seedlings to 200 mM NaCl reduced collar diameter in control seedlings by 31.12%, from 6.07 to 4.18 mm. Under saline conditions, collar diameter ranged from 5.14 to 5.48 mm in inoculated seedlings. The highest value was observed with *P. flexa* C5 (5.48 mm), corresponding to a 30.98% increase relative to the salt control, followed by *P. flexa* S6 (24.95%), *P. flexa* R7 (23.57%), *B. subtilis* C7 (23.33%), and *B. subtilis* R3 (22.95%) (Fig. 7B).

### Effect of bacterial inoculation on the number of leaves of tomato seedlings under salt stress

The results revealed a highly significant effect of treatments on leaf number [F (11,108) = 25.80, *p* < 0.0001]. However, no significant differences were detected among treatments within the same salinity level. Under non-saline conditions, the number of leaves ranged from 7.2 to 8.0. *B. subtilis* R3 exhibited the highest value of 8 leaves, corresponding to an 11.1% increase compared to the uninoculated control (7.2 leaves). *P. flexa* C5, *B. subtilis* C7, *P. flexa* S6, and *P. flexa* R7 also show an increase in the number of leaves. Exposure to 200 mM NaCl reduced the average number of leaves by 22.2%, from 7.2 to 5.6 in the control treatment. Under saline conditions, inoculated seedlings produced between 5.8 and 6.1 leaves. The highest value was observed with *P. flexa* S6 (6.1 leaves), corresponding to an 8.9% increase relative to the salt control. *P. flexa* R7 and *B. subtilis* C7 also increased leaf number (Fig. 7C).

### Effect of bacterial inoculation on the root length of tomato seedlings under salt stress

**Fig. 9:**
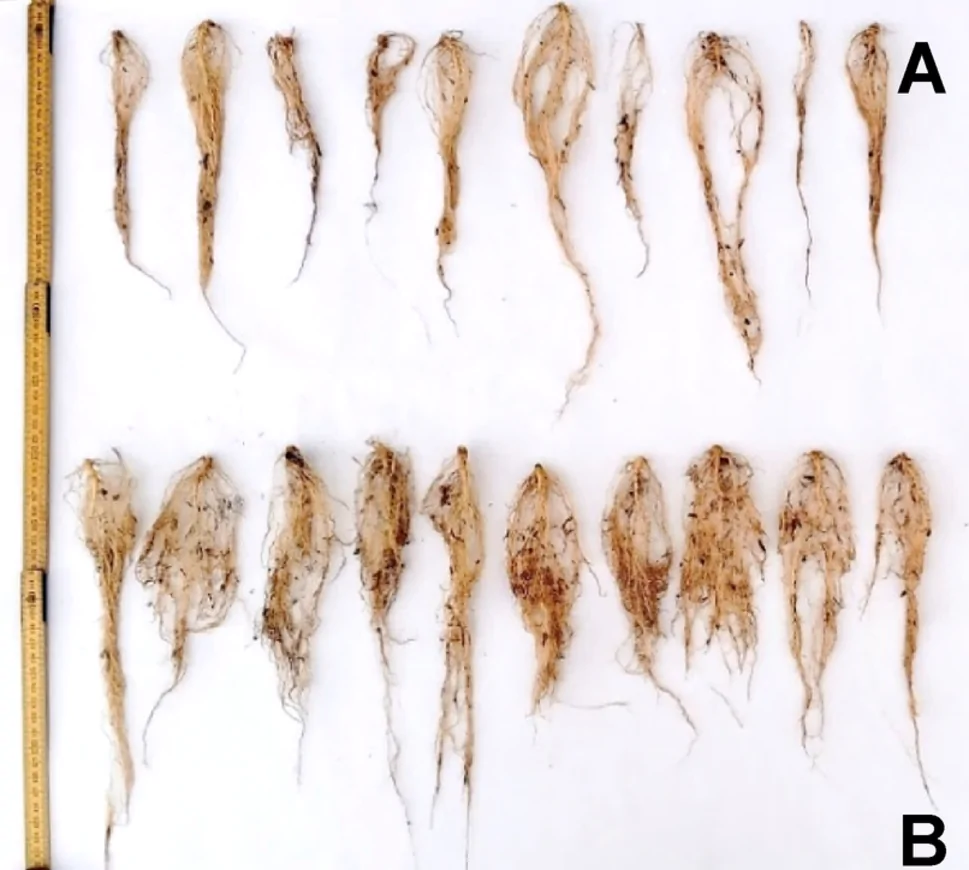
Effect of *P. flexa* C5 inoculation on root length of six-week-old tomato seedlings under 200 mM NaCl. **A** salt control (200 mM NaCl); **B** seedlings inoculated with *P. flexa* C5 under 200 mM NaCl

The results revealed a highly significant effect of treatments on root length [F (11,108) = 6.561, *p* < 0.0001]. In the absence of salt stress, mean root length ranged from 24.6 to 33.3 cm. *P. flexa* C5 exhibited the highest root length of 33.3 cm, corresponding to a 21.8% increase compared to the uninoculated control (27.35 cm). *B. subtilis* R3 and *P. flexa* R7 also show an improvement of this parameter, reaching mean root lengths of 31.8 and 28.75 cm, respectively. Exposure to 200 mM NaCl reduced mean root length by 22.1%, from 27.35 cm in the no-salt control to 21.3 cm in the salt control. Under saline conditions, root length ranged from 21.56 to 26.75 cm in inoculated seedlings. The highest root length was observed in seedlings inoculated with *P. flexa* C5 (26.75 cm), representing a 25.6% increase relative to the salt control. *P. flexa* R7 (24.95 cm) and *B. subtilis* R3 (23.5 cm) also increased root length (Figs. 7D and 9).

### Effect of bacterial inoculation on the fresh leaf weight of tomato seedlings under salt stress

The results revealed a highly significant effect of treatments on fresh leaf weight [F (11,108) = 61.63, *p* < 0.0001]. However, no significant differences were detected among treatments under saline conditions (200 mM NaCl). Under non-saline conditions, mean fresh leaf weight ranged from 32.83 to 38.00 g. *P. flexa* C5 exhibited the highest fresh leaf weight of 38.00 g, corresponding to a 15.5% increase compared to the uninoculated control (32.89 g). *B. subtilis* R3 (36.01 g) and *P. flexa* S6 (34.82 g) had higher fresh leaf weights than the control. Exposure to 200 mM NaCl reduced mean fresh leaf weight by 49.7%, from 32.89 g in the no-salt control to 16.54 g in the salt control. Under saline conditions, fresh leaf weight ranged from 17.58 to 20.54 g in inoculated seedlings. The highest fresh leaf weight was observed with *P. flexa* R7 (20.54 g), representing a 24.2% increase compared to the salt control. *P. flexa* C5 (20.12 g) and *B. subtilis* C7 (19.87 g) also increased fresh leaf weight under salt stress (Fig. 7E).

### Effect of bacterial inoculation on the fresh root weight of tomato seedlings under salt stress

The results revealed a highly significant effect of treatments on fresh root weight [F (11,108) = 16.04, *p* < 0.0001]. Under non-saline conditions, mean fresh root weight ranged from 4.51 to 8.05 g. *B. subtilis* R3 exhibited the highest fresh root weight (8.05 g), corresponding to a 78.5% increase compared to the uninoculated control (4.51 g). *B. subtilis* C7 (6.30 g), *P. flexa* R7 (6.29 g), *P. flexa* S6 (6.28 g), and *P. flexa* C5 (6.02 g) had higher fresh root weights than the control. Exposure to 200 mM NaCl reduced mean fresh root weight by 65.1%, from 4.51 g in the no-salt control to 1.57 g in the salt control. Under saline conditions, fresh root weight ranged from 2.92 to 4.21 g in inoculated seedlings. The highest fresh root weight was observed with *P. flexa* S6 (4.21 g), corresponding to a 167.5% increase compared to the salt control. *P. flexa* R7 (4.01 g) and *B. subtilis* C7 (3.89 g) also increased fresh root weight under salt stress (Fig. 7F).

### Effect of bacterial inoculation on the dry leaf weight of tomato seedlings under saline stress

The results revealed a highly significant effect of treatments on leaf dry weight [F (11,108) = 141.5, *p* < 0.0001]. Under non-saline conditions, mean leaf dry weight ranged from 5.65 to 7.26 g. *B. subtilis* R3 exhibited the highest leaf dry weight (7.26 g), corresponding to a 28.5% increase compared to the uninoculated control (5.65 g). *P. flexa* C5 (7.25 g), *P. flexa* S6 (7.07 g), and *B. subtilis* C7 (6.85 g) also produced higher leaf dry weights than the control. Exposure to 200 mM NaCl reduced mean leaf dry weight by 70.1%, from 5.65 g in the no-salt control to 1.69 g in the salt control. Under saline conditions, leaf dry weight ranged from 2.37 to 2.93 g in inoculated seedlings. The highest leaf dry weight was observed with *P. flexa* R7 (2.93 g), corresponding to a 73.0% increase compared to the salt control. *P. flexa* C5 (2.85 g), *B. subtilis* C7 (2.85 g), and *P. flexa* S6 (2.56 g) also increased leaf dry weight under salt stress (Fig. 7G).

### Effect of bacterial inoculation on the root dry weight of tomato seedlings under salt stress

The results revealed a highly significant effect of treatments on root dry weight [F (11,108) = 44.36, *p* < 0.0001]. However, no significant differences were detected among treatments under saline conditions (200 mM NaCl). Under non-saline conditions, mean root dry weight ranged from 1.03 to 1.52 g. *P. flexa* R7 exhibited the highest root dry weight (1.52 g), corresponding to a 47.0% increase compared to the uninoculated control (1.03 g). *P. flexa* C5 (1.32 g), *B. subtilis* R3 (1.31 g), and *B. subtilis* C7 (1.27 g) also produced higher root dry weights than the control. Exposure to 200 mM NaCl reduced mean root dry weight by 70.5%, from 1.03 g in the no-salt control to 0.31 g in the salt control. Under saline conditions, root dry weight ranged from 0.43 to 0.59 g in inoculated seedlings. The highest root dry weight was observed with *B. subtilis* C7 (0.59 g), corresponding to a 94.9% increase compared to the salt control. *P. flexa* C5 (0.57 g) and *P. flexa* R7 (0.56 g) also increased root dry weight under salt stress (Fig. 7H).

### Proline content of tomato plants under saline stress after bacterial inoculation

**Fig. 10:**
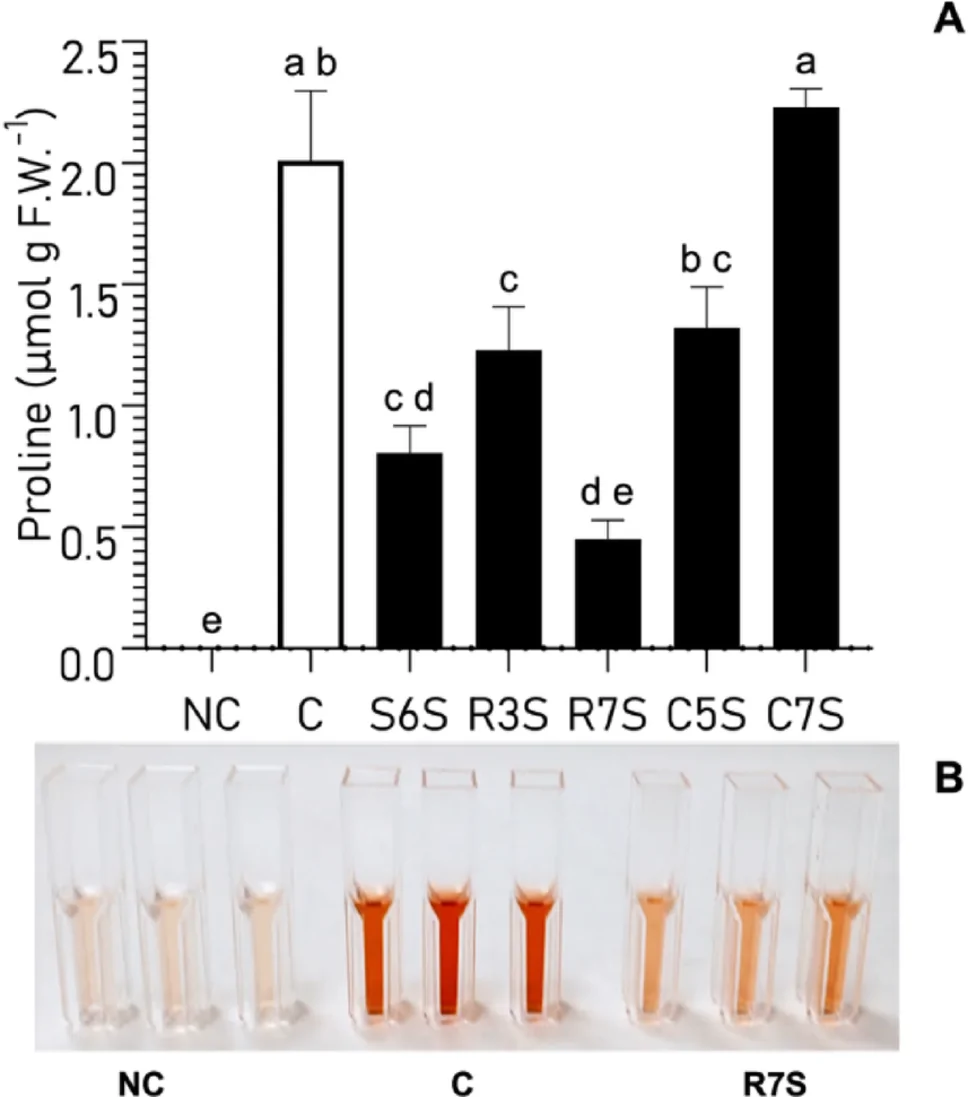
Effect of bacterial inoculation on proline accumulation in tomato plants subjected to salt stress (200 mM NaCl): **A** proline content under the different treatments and **B** representative samples obtained during the proline assay. Values followed by the same letter are not significantly different (*p* > 0.05). NC: no-salt control; C: salt control (200 mM NaCl); S6S: *P. flexa* S6 + 200 mM NaCl; R3S: *B. subtilis* R3 + 200 mM NaCl; R7S: *Priestia flexa* R7 + 200 mM NaCl; C5S: *P. flexa* C5 + 200 mM NaCl; C7S: *B. subtilis* C7 + 200 mM NaCl

The results revealed a highly significant effect of treatments on proline content (F = 27.05, *p* < 0.0001). No proline accumulation was detected in the no-salt control (T, 0 mM NaCl), for which the mean proline content was 0.000 (Fig. 10). In contrast, exposure to 200 mM NaCl resulted in substantial proline accumulation in the salt control (S), reaching a mean value of 2.010. Among the inoculated treatments, the lowest proline content was observed with *P. flexa* R7 (0.450), followed by *P. flexa* S6 (0.804), *B. subtilis* R3 (1.228), and *P. flexa* C5 (1.320). In contrast, *B. subtilis* C7 exhibited the highest proline content (2.228), exceeding that recorded in the salt control. Relative to the salt control, proline content was reduced by approximately 78% in plants inoculated with *P. flexa* R7, 60% with *P. flexa* S6, 39% with *B. subtilis* R3, and 34% with *P. flexa* C5.

## Discussion

Salinity is one of the main abiotic stressors limiting crop productivity (Adeleke et al. 2024). In plants, excess salts cause osmotic stress, ionic toxicity, and nutritional imbalances that disrupt water uptake, photosynthesis, and cellular metabolism, leading to reduced growth and productivity (Esan and Tounsi 2026). PGPB represent a promising biological approach to mitigating the effects of salinity stress, owing to their ability to improve mineral nutrition, modulate root development, and strengthen plant adaptive mechanisms to osmotic stress (Ren et al. 2026; Wang and Xu 2026). In this context, this study evaluated the potential of halotolerant bacteria associated with *O. dillenii* to improve growth and salt stress tolerance in tomatoes.

This study revealed that several bacterial strains associated with *O. dillenii* could grow under high NaCl concentrations. Nearly 52.38% of the isolates grew at NaCl concentrations exceeding 1.5 M, and six strains tolerated up to 2.5 M. This high level of salt tolerance is consistent with the ecological origin of the isolates, as microorganisms from coastal environment naturally show high osmotic stress tendencies. Several recent studies have shown that plants living in saline or arid habitats preferentially harbor microbial communities enriched with halotolerant properties that support their adaptability and survival under high salt concentrations (Ramasamy and Mahawar 2023; Li et al. 2025; Jadhav et al. 2026). The predominance of *P. flexa* among the most tolerant bacterial isolates is noteworthy, as four of the six strains capable of growing up to 2.5 M NaCl (≈ 14.6% NaCl) belonged to this species. Soto-Varela et al. (2024) reported that an endophytic strain of *P. flexa* isolated from an *Avicennia germinans* mangrove was capable of growing in the presence of 12.5% NaCl and possessed several mechanisms that facilitate its adaptation to extreme salinity conditions. The tolerance observed in *B. amyloliquefaciens* R4 and C8 is also consistent with previous studies describing this species as highly tolerant to a range of abiotic stresses (Wu et al. 2024; Bisht et al. 2025).

Halotolerant bacteria are capable of accumulating compatible osmoprotectants, such as proline, glycine betaine, and trehalose; maintaining ionic homeostasis via specialized transport systems; and producing exopolysaccharides that help reduce the effects of osmotic stress (Kumar et al. 2023; Ameen et al. 2024). However, the absence of growth observed at 3 M NaCl indicates the existence of a physiological limit common to all isolates. Several studies have also shown that the growth of halotolerant bacteria decreases sharply as salinity approaches hypersaline conditions (Li et al. 2025; Jadhav et al. 2026). In this study, exposure of tomato seeds to 120 mM NaCl reduced the germination rate to 50% and lowered the seedling vigor index, confirming the sensitivity of the germination stage to salt stress. This reduction is primarily attributed to the decrease in the osmotic potential of the medium, which limits water uptake by the seed and slows the activation of the metabolic processes necessary for the resumption of embryonic growth (Manono 2025). Furthermore, the accumulation of Na⁺ and Cl⁻ ions can cause ionic imbalances, enzymatic disturbances, and oxidative stress that directly affect germination and the initial growth of seedlings. These mechanisms have been widely described as the primary causes of germination inhibition under saline conditions (Li et al. 2025; Manono 2025).

However, seed biopriming with halotolerant bacterial strains significantly reduced the deleterious effects of NaCl. The strains *P. flexa* S6 and *P. flexa* C5 increased the germination rate by 50–75%, while *P. flexa* S6 and *P. flexa* R7 induced the greatest increases in the vigor index. These results corroborate numerous studies showing that early seed inoculation with growth-promoting bacteria improves the speed, uniformity, and percentage of germination under stressful conditions (González-Cobo et al. 2024; Gupta et al. 2025). Biopriming also promotes early colonization of emerging tissues, allowing beneficial bacteria to interact with the plant from the earliest stages of development (Fiodor et al. 2023; Adeboye et al. 2025).

Rather (2025) reported that beneficial endophytes can enhance seed germination and early seedling establishment through improved nutrient acquisition, phytohormone production and stress adaptation mechanisms, thereby increasing plant resilience during the earliest developmental stages. An important observation was the absence of a significant effect of the strains on hypocotyl and radicle length under non-saline conditions, whereas highly significant differences were observed under 120 mM NaCl. This observation indicates that the selected bacteria do not simply act as general growth stimulators but primarily exert their effect when the plant is subjected to salt stress. Furthermore, the greatest effects were consistently observed with the strains *P. flexa* R7, S6, and C5, which significantly improved hypocotyl and radicle elongation compared to the stressed control. This predominance suggests that *P. flexa* may possess effective mechanisms for maintaining the growth of juvenile tissues under unfavorable osmotic conditions. The fact that these strains were also among the most halotolerant isolates in the initial screening reinforces the hypothesis that their ability to withstand high concentrations of NaCl constitutes a decisive advantage for maintaining plant growth under salinity stress (Lenka 2026).

The application of 200 mM NaCl significantly affected the growth of tomato plants by reducing plant height, stem diameter at the collar, number of leaves, root length, and both above-ground and root biomass. The most significant reductions were observed in fresh and dry root weights, which decreased by 65.1% and 70.5%, respectively, confirming the root system’s high sensitivity to salt stress. However, bacterial inoculation significantly mitigated these negative effects.

Although some inoculated strains showed numerical increase in plant height under salinity stress, these differences were not statistically significant. This result suggests that plant height was less responsive to bacterial inoculation than other growth parameters. In contrast, significant improvements were observed for collar diameter, root length, as well as fresh and dry biomass, indicating that the beneficial effects of the selected strains were primarily expressed through enhanced root development and greater biomass accumulation rather than through increased shoot elongation. Under high salinity conditions, maintenance of root growth represents an important adaptive advantage for water and nutrient acquisition, thereby contributing to the maintenance of plant performance under stress (Holz et al. 2024). This response may explain the improvements observed in several growth parameters despite the absence of a significant effect on plant height.

The greatest improvements observed with *P. flexa* strains, particularly S6, C5, and R7, improved several growth parameters despite the severity of the applied stress. These results were consistent with the findings of Arminjon and Lefort (2025), who reported a significant reduction in tomato growth under 200 mM NaCl, i.e. a decrease in the fresh and dry above-ground biomass by 53.4% and 67.9%, respectively, which is similar to the values obtained in this study for fresh and dry leaf weights. However, while the improvements reported by these authors mainly concerned above-ground biomass, our results place greater emphasis on the maintenance of root development. In particular, *P. flexa* S6 increased fresh root weight by 167.5%, while *P. flexa* C5 improved root length by 25.6%, suggesting an enhanced capacity to sustain root development and potentially improve water acquisition under severe salinity stress. Similar observations have been reported by Patani et al. (2023), who showed that several *Bacillus* species enhanced tomato growth under saline conditions. However, the authors’ study focused on salinity levels of approximately 40–45 mM NaCl, a stress level significantly lower than those applied in this study. Despite this much higher stress level, the improvements observed with *P. flexa* S6 in fresh root biomass and with *P. flexa* C5 in root length remain substantial. These results suggest that bacteria associated with *O. dillenii* can contribute significantly to the maintenance of root development under salinity levels that may be detrimental to plant growth. A study evaluating the efficacy of PGPR under salinity levels reaching 207 mM NaCl has reported a decrease in bacterial effects on plant growth as salt concentration increased (Yahyaoui et al. 2024). In this study, the *P. flexa* strains S6, C5, and R7 maintained significant beneficial effects at 200 mM NaCl, particularly on root parameters and biomass. The observed effects of the *P. flexa* strains may be related to their functional traits. Strain C5 has the most comprehensive PGP profile among the three selected isolates, combining the production of exopolysaccharides and IAA with nitrogen fixation and phosphate solubilization (Brun et al. 2026). This profile explains its remarkable effects on collar diameter and root length. Exopolysaccharide production may additionally improve salinity tolerance by increasing water retention around roots and reducing Na⁺ toxicity in the rhizosphere, whereas IAA production directly stimulates root elongation and root hair development (Tariq et al. 2025). The results obtained are consistent with the findings of Lin et al. (2022), who showed that a *Priestia* strain with similar activities effectively stimulated root development and plant growth. The beneficial effects observed in *P. flexa* strains are likely multifactorial and may involve phytohormone production, exopolysaccharide synthesis, improved nutrient acquisition, osmotic regulation and oxidative stress mitigation. According to Fagnano et al. (2025), the development of next-generation microbial biostimulants should rely on the selection and combination of functionally complementary endophytes capable of enhancing plant adaptation to abiotic stresses.

Saline stress caused a significant accumulation of proline in the control plants exposed to 200 mM NaCl. Bacterial inoculation reduced this accumulation, with decreases of 78%, 60%, and 34% for *P. flexa* R7, S6, and C5, respectively. Proline is generally considered an indicator of osmotic stress. Thus, the decrease observed in inoculated plants suggests a reduction in the intensity of stress perceived by the plant. Similar reductions in stress-related metabolites have been reported in plants inoculated with beneficial endophytes, where improved osmotic adjustment and antioxidant protection decrease the requirement for proline accumulation under salinity stress (Tariq et al. 2025). The reduced proline accumulation observed in inoculated plants may result from the ability of PGPR to alleviate salt-induced stress through improved ion homeostasis, modulation of osmotic stress responses, and enhancement of antioxidant defense systems. By reducing the severity of physiological stress, these mechanisms may decrease the requirement for excessive proline accumulation as a protective osmolyte (Mellidou et al. 2021; Naseri et al. 2022). This response was most evident in *P. flexa* R7, which also exhibits the highest above-ground biomass. Hua et al. (2025), who observed an increase in proline following inoculation with *P. megaterium.* Our results suggest a reduced requirement for proline accumulation, which were more consistent with the observations of Hwang et al. (2022), who reported the ability of *Priestia* strain to reduce salinity-induced oxidative damage. In this context, the decrease in proline observed in R7 may reflect an improvement in plant physiological status rather than a simple change in osmotic metabolism (Avendaño et al. 2025). Hence, genomic or transcriptomic approaches would be necessary to determine whether the observed performance is associated with genes involved in osmotic regulation, ion transport, exopolysaccharide synthesis, or other stress response mechanisms.

## Conclusion

This study demonstrates the potential of halotolerant bacteria associated with *O. dillenii* to alleviate the adverse effects of salinity stress in tomato. Among the 21 isolates evaluated, several strains exhibited high tolerance to elevated NaCl concentrations and displayed traits associated with plant growth promotion. Bacterial inoculation improved seed germination, seedling vigor, and plant growth under saline conditions. The most pronounced effects were observed with *P. flexa* strains S6, C5, and R7. Under 200 mM NaCl, these strains contributed to the maintenance of several growth parameters, including root development, biomass production, and physiological performance. Strain S6 was particularly effective in increasing fresh root biomass, C5 enhanced root development and collar diameter, whereas R7 promoted aboveground biomass accumulation while markedly reducing proline content.

These findings highlight *O. dillenii* as a valuable and underexplored reservoir of salt-adapted bacteria with promising potential for the development of microbial biostimulants for saline agriculture. However, the physiological and molecular mechanisms underlying the observed responses were not investigated in detail. Further studies integrating genomic, transcriptomic, and metabolomic approaches are needed to improve our understanding of the mechanisms underlying the salt tolerance-promoting effects observed in the most effective strains.

## Data Availability

Data is contained within the article or supplementary material. The datasets generated during the current study are available from the corresponding author on reasonable request.
